# Hemifusion in Synaptic Vesicle Cycle

**DOI:** 10.3389/fnmol.2017.00065

**Published:** 2017-03-16

**Authors:** Dae-Hyuk Kweon, Byoungjae Kong, Yeon-Kyun Shin

**Affiliations:** ^1^Department of Genetic Engineering, College of Biotechnology and Bioengineering, Sungkyunkwan UniversitySuwon, South Korea; ^2^Department of Biochemistry, Biophysics and Molecular Biology, Iowa State UniversityAmes, IA, USA

**Keywords:** SNARE, membrane fusion, hemifusion, fusion pore, transmembrane domain

## Abstract

In the neuron, early neurotransmitters are released through the fusion pore prior to the complete vesicle fusion. It has been thought that the fusion pore is a gap junction-like structure made of transmembrane domains (TMDs) of soluble N-ethylmaleimide-sensitive-factor attachment protein receptor (SNARE) proteins. However, evidence has accumulated that lipid mixing occurs prior to the neurotransmitter release through the fusion pore lined predominantly with lipids. To explain these observations, the hemifusion, a membrane structure in which two bilayers are partially merged, has emerged as a key step preceding the formation of the fusion pore. Furthermore, the hemifusion appears to be the bona fide intermediate step not only for the synaptic vesicle cycle, but for a wide range of membrane remodeling processes, such as viral membrane fusion and endocytotic membrane fission.

## Introduction

**Figure 1:**
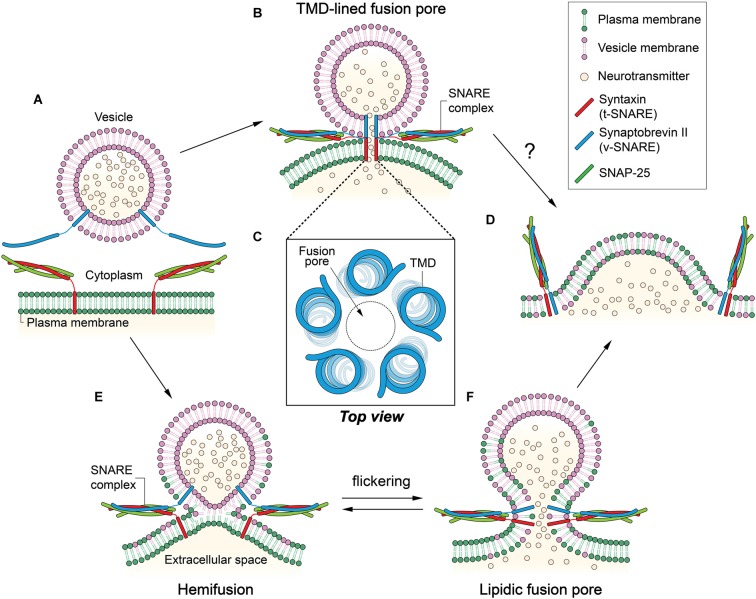
**Two contradicting mechanisms for the formation of fusion pore. (A)** Synaptobrevin-2 (Syb2) is anchored to a synaptic vesicle, and the t-soluble N-ethylmaleimide-sensitive-factor attachment protein receptor (SNARE) complex composed of syntaxin-1 (Stx1) and SNAP-25 is anchored to the plasma membrane. Membrane fusion is mediated by the formation of the SNARE four-helix bundle. **(B)** In the transmembrane domain (TMD)-lined fusion pore model, a gap junction-like fusion pore is formed by TMDs of SNARE proteins. Two hemi-pores, one formed by Stx1 TMDs and the other by Syb2 TMDs, dock to constitute a long pore through which neurotransmitters can be released. **(C)** Five to eight TMDs may form a pore in the center. **(D)** The TMD-lined pore model cannot explain how fusion between the two membranes is achieved or how the small fusion pore eventually dilates to complete fusion reaction. **(E)** In the hemifusion, outer leaflets are merged while inner leaflets remain separate. **(F)** A lipidic fusion pore is formed by inner leaflet mixing. This small fusion pore is in equilibrium with the hemifusion, which may result in flickering. The small fusion pore would eventually dilate to complete membrane fusion.

Neurotransmitter release from the neuron requires fusion of vesicles to the plasma membrane. However, the bilayer structure is highly stable; thus, two bilayers normally do not fuse spontaneously. It is thought that conserved soluble N-ethylmaleimide-sensitive-factor attachment protein receptor (SNARE) proteins mediate synaptic vesicle fusion (Söllner et al., 1993a,b). Three SNARE proteins involved in neuroexocytosis are synaptobrevin-2 (Syb2, also called VAMP2), syntaxin-1 (Stx1), and SNAP-25. Syb2 is the vesicle membrane (v-) SNARE of 116-amino acids with a single transmembrane domain (TMD). Stx1 is the 288-amino acid protein attached to the plasma membrane, likewise with a single TMD. SNAP-25 has lipid anchors in the plasma membrane and forms the target membrane (t)-SNARE complex with Stx1 (Figure 1A). Cognate SNARE motifs that protrude respectively from two membranes assemble to form a parallel four-helix bundle (Poirier et al., 1998; Sutton et al., 1998) that drives apposition and subsequent fusion of two membranes (Weber et al., 1998). It is believed that SNARE proteins progressively zipper from the membrane-distal N-terminal region toward the membrane-proximal C-terminal region (Fiebig et al., 1999; Chen et al., 2001; Melia et al., 2002; Matos et al., 2003; Sorensen et al., 2006; Ellena et al., 2009; Gao et al., 2012; Lou and Shin, 2016).

A crucial question towards elucidating the pathway of membrane fusion is what happens to the bilayers when two membranes merge. Obviously, our interests are on the role that SNARE TMDs play in fusion because they are located at or near the epicenter of membrane fusion. There are two proposed fusion pathways that predict two very different roles of the TMDs. The first model (Han et al., 2004), which is primarily based on electrophysiological measurements, argues that the TMDs serve as the principal building block of the fusion pore that is considered a bona fide intermediate for synaptic vesicle fusion. In stark contrast, the second model (Xu et al., 2005), largely based on spectroscopic and structural studies, predicts that lipids are major building blocks that build the “hemifusion” and the fusion pore. In the latter, TMDs play a supporting role either as membrane anchors for the soluble SNARE complex or as mechanical levers working at the periphery.

Although the TMD-lined fusion pore model was developed earlier to explain electrophysiological results, much evidence has now accumulated in favor of membrane fusion through hemifusion for SNARE-dependent vesicle fusion. Furthermore, the hemifusion might be a common intermediate shared by many membrane fusion and fission systems including viral-cell membrane fusion (Melikyan et al., 1995; Chernomordik et al., 1998; Chernomordik and Kozlov, 2003) and endocytotic membrane fission (Antonny et al., 2016).

## TMD-Lined Fusion Pore

There is overwhelming evidence that vesicle fusion transits through the fusion pore (Neher and Marty, 1982; Breckenridge and Almers, 1987; Chow et al., 1992; Alvarez de Toledo et al., 1993; Lollike et al., 1995), a metastable intermediate with a small aqueous opening through two apposed membranes. In this state, the vesicle is expected to remain nearly intact except a pore that continues through the plasma membrane. The size of the fusion pore is estimated to be similar to those of large ion channels. Some small but detectable amounts of neurotransmitters can pass through the fusion pore. Electrophysiological measurements of fusion pore conductance revealed that the fusion-pore diameter remains <3 nm and can persist for a few seconds (Albillos et al., 1997).

In an earlier study, no flow of lipids was observed at the stage of the fusion pore formation (Klyachko and Jackson, 2002). Combining the electrophysiological data, one could envision a gap junction-like fusion pore in which SNARE complex formation brings about docking of two hemi-pores made of v- and t-SNARE TMDs, respectively, to produce a longer, complete pore that continues through the two membranes (Figure 1B).

Experimental evidence that supports such a protein-lined fusion pore was obtained by amperometric study of Stx1 mutants (Han et al., 2004). Han et al. (2004) found that Trp mutations at the putative pore-lining residues in the TMD interfere with the release of the neurotransmitters, consistent with the model. Based on the conductance measurements, they modeled a helix-lined pore consisting of 5–8 TMDs with a 0.5 nm pore at the center (Figure 1C).

Although the data qualitatively agree with the model, one caveat is that the impedance of the release due to the Trp mutation is much lower than expected. There is only a 20%–30% decrease in the release with the mutation. In reality, one would expect that 5–8 Trp residues at the same layer in the 0.5-nm-diameter pore would completely fill the space, which would allow little passage of neurotransmitters through the pore. On the other hand, two membranes are still fully separated in this stage, and the model does not explain how structurally and energetically the two bilayers eventually merge to complete the fusion reaction (Figure 1D; Chernomordik and Kozlov, 2003).

Recently, Cys-scanning experiments showed that V101 and I105 in the TMD of Syb2 might line the fusion pore (Bao et al., 2016). However, the small nanodisc of ~6 nm diameter used in the Cys-scanning experiments, contained as few as two copies of Syb2. The reality is that two copies of the TMD would not be able to form a TMD-lined pore. Alternatively, an idea that the fusion pore may be both lipidic and proteinaceous was suggested on the basis of an amperometry study of chromaffin cells expressing C-terminal truncation mutants of SNAP-25 (Fang et al., 2008). The layout of TMDs in this model is however purely imaginary with little experimental support.

The early result that there was no lipid flow at the fusion pore stage is an important basis for developing a gap junction-like fusion model. However, it is not unusual to have poor lipid mixing when the protein density is high, as is shown for influenza virus-cell fusion (Chernomordik et al., 1998). Thus, one could argue that limited lipid mixing might not be a sufficient condition for a gap junction-like fusion pore. After all, the TMD might not be a required component to complete membrane fusion. In fact, TMD-less, lipid-anchored SNAREs are sufficient for healthy neurotransmitter release in the neuron (Zhou et al., 2013), vacuole fusion in yeast (Xu et al., 2011), and proteoliposome fusion *in vitro* (McNew et al., 2000), raising concerns on the validity of the TMD-lined fusion pore model.

## Hemifusion

Let us now consider alternative possibilities to the TMD-lined fusion pore. For membrane fusion between influenza virus and red blood cells, Kemble et al. (1994); Melikyan et al. (1995) made a seminal discovery that uncovered a lipid-dominant intermediate in membrane fusion (Kemble et al., 1994; Melikyan et al., 1995). The authors found that a GPI-anchored hemagglutinin mutant arrests membrane fusion at the intermediate state in which lipid mixing is allowed while content mixing is not.

The observation by Kemble et al. (1994); Melikyan et al. (1995) appears to be consistent with a theoretical model for membrane fusion, developed for protein-free fusion of two lipid bilayers, on the basis of the lipid-stalk intermediate (Kozlov et al., 1983). We call the half-merged state in which inner leaflets are intact while the outer leaflets are merged the “hemifusion”. The predicted hemifusion was later imaged experimentally with x-ray crystallography for the macroscopically aligned, protein-free multi-bilayer (Yang and Huang, 2002).

At hemifusion, lipid mixing may be allowed through outer leaflets (Figure 1E). However, lipid mixing through inner leaflets is also possible because the hemifusion could be in equilibrium with small fusion pores that flicker (Figure 1F; Chanturiya et al., 1997). In some cases, the hemifusion diaphragm can be formed via expansion of the hemifusion into a large area (Hernandez et al., 2012).

The idea that intracellular membrane fusion might transit through a structure of the curved membrane was percolated through the observation that the exogenously added lysolipids impair exocytosis in cells (Chernomordik et al., 1993). It has been thought that the molecular shape of a lipid correlates with the effective spontaneous curvature. While cylindrical phosphatidylcholine forms the almost flat monolayer, cone-shaped phosphatidylethanolamine and diacylglycerol bulge in the direction of the acyl chains and favor the net negative curvature. In contrast, lysolipids such as lysophosphatidylcholine and polyphosphoinositides are inverted cone-shaped molecules with large polar heads and thin acyl chain tails that prefer the positively curved membrane to the negatively curved one. Net negative membrane curvature happens to be a characteristic feature of the hemifusion (Chernomordik and Kozlov, 2008).

## Hemifusion in SNARE-Dependent Membrane Fusion

The first direct experimental evidence of hemifusion in SNARE-dependent fusion was from *in vitro* fusion assays employing SNARE-reconstituted proteoliposomes (Lu et al., 2005; Xu et al., 2005). The fluorescence measurements showed that lipids in the outer leaflets mix faster than those in the inner leaflets. Furthermore, the fusion reaction between single proteoliposomes, studied with total internal reflection microscopy, showed distinct steps that reflected the hemifusion (Yoon et al., 2006).

Hemifusion has also been identified in fusion of vacuoles from yeast (Jun and Wickner, 2007) as well as in Ca^2+^-triggered exocytosis in chromaffin cells (Wong et al., 2007). Additionally, the hemifusion was observed in cell-cell fusion by flipped SNAREs (Giraudo et al., 2006) and in fusion between a SNARE-reconstituted nanodisc and a liposome (Shi et al., 2012), although it is unclear whether the hemifusion observed here is a dead-end product or not. Recently, signals reflecting hemifusion have been detected in the force measurement in SNARE-mediated fusion of proteoliposome to the supported bilayer (Oelkers et al., 2016). Furthermore, a hemifusion structure was visualized in the study of neurons with electron tomography at low resolution (Zampighi et al., 2006).

Although there is overwhelming evidence that the hemifusion does exist in SNARE-dependent membrane fusion, there is still a controversy concerning whether the hemifusion is on- or off-pathway in Ca^2+^-triggered exocytosis. In fact, Diao et al. (2012) raised the possibility that hemifusion might be an off-pathway intermediate in Ca^2+^-triggered exocytosis (Diao et al., 2012). In their *in vitro* experiments, full fusion has occurred within the pool of un-hemifused proteoliposomes, although it is still possible that the hemifusion is too short-lived to be detected under their experimental conditions. Alternatively, in some limited cases, the hemifusion can expand into the hemifusion diaphragm, which may not progress readily to full fusion (Hernandez et al., 2012).

There is still controversy on whether the TMDs are even required in the neuro-exocytosis. While Zhou et al. (2013) found that GPI-anchored Syb2 fully supports the neurotransmitter release (Zhou et al., 2013). Han et al. (2004) showed that various lipid-anchored Syb2 variants provide little support of exocytosis (Chang et al., 2016). Although SNARE TMDs may not be essential, it turns out that they play important, active roles in modulating SNARE-dependent membrane fusion. For example, Shin et al. (2014) have shown that cholesterol could change the conformation of the dimeric Syb2 TMD to be favorable for membrane fusion (Tong et al., 2009). A simulation study found that the SNARE TMDs might play a role in initiating fusion by distorting the lipid packing of the outer leaflets (Risselada et al., 2011). It is also shown that the conformational flexibility of the Syb2 TMD might lower the negative membrane curvature within the outer leaflet of the fusion pore neck to facilitate pore expansion (Dhara et al., 2016).

## Coupling of SNARE Zippering to the Hemifusion

SNARE motifs assemble into a stable, parallel, four-stranded coiled coil. The SNARE complex is made of 15 (numbered −7 to +8) hydrophobic layers and one ionic zeroth layer at the center. There is evidence that SNARE motifs zipper from the membrane-distal N-terminal region towards the membrane-proximal C-terminal region (Fiebig et al., 1999; Chen et al., 2001; Melia et al., 2002; Sorensen et al., 2006; Su et al., 2008; Ellena et al., 2009). After transitioning through intermediate structures that might serve as structural platforms for interactions with other accessory proteins, the SNARE complex ends up at a *cis*-conformation representing the post fusion state in which TMDs of Stx1 and Syb2 reside in the same membrane. Prior to *cis*-complex formation, partial complexes are present, of which the degree of zippering was only recently revealed. It was found, from single-molecule force measurements, that SNARE complex formation may occur in at least two steps, with a pivot at the conserved “zeroth” layer in the middle (Li et al., 2007; Gao et al., 2012; Min et al., 2013; Shin et al., 2014; Zorman et al., 2014). Now, given that SNARE zippering drives apposition of two membranes, it must be determined at what stage of SNARE zippering hemifusion occurs. Precise mapping of the degrees of SNARE zippering to specific stages of membrane fusion is prerequisite to the understanding of the mechanism of membrane fusion.

**Figure 2:**
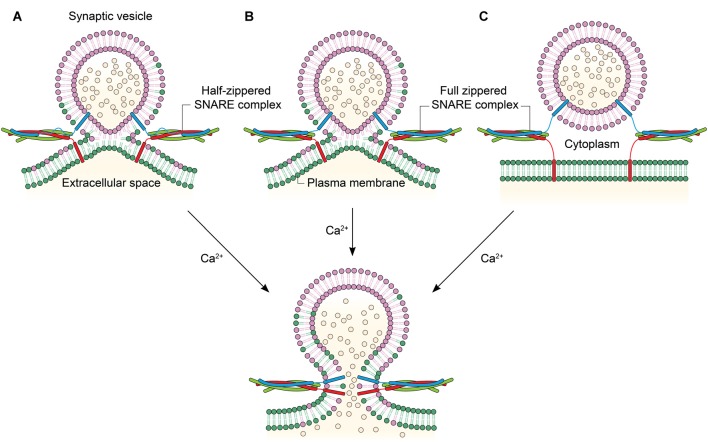
**Linking the degree of zippering to the hemifusion. (A)** Hemifusion is achieved by only the zippering of the N-terminal half of SNARE motifs. Ca^2+^ induces fusion pore opening by enabling full SNARE zippering through the C-terminal half of SNARE motifs and TMDs. **(B)** Full SNARE zippering induces hemifusion. A fusion pore can be opened by Ca^2+^. **(C)** Full SNARE zippering is required to dock vesicles to the plasma membrane. In this case, Ca^2+^ may drive hemifusion and subsequent full fusion almost simultaneously to achieve fast exocytosis.

An immediate, related question in the field has been if membranes are hemifused before the Ca^2+^ influx. Hemifusion induced by SNARE complex formation before Ca^2+^ was reported (Figure 2B), for the first time, by Schaub et al in their *in vitro* investigation of the regulation of SNARE-dependent vesicle fusion by Syt1 and complexin (Schaub et al., 2006). This result was verified with the observations that the hemifusion is a stable intermediate of exocytosis in neuronal cells *in vivo* (Wong et al., 2007) and that two membranes may be hemifused before Ca^2+^ influx (Zampighi et al., 2006). In contrast, it has been proposed that syt1 and Ca^2+^ play a role in driving lipid stalk formation (Martens et al., 2007; Hui et al., 2009), indicating that lipid mixing or stalk formation occur after Ca^2+^ influx (Figure 2C). However, this model has not been verified with *in vivo* results.

Interestingly, it has been recently shown that SNARE zippering of only the N-terminal half could drive hemifusion (Figure 2A; Yang et al., 2010). SNARE complex formation can be arrested at the half-zippered state by a flavonoid myricetin, and it is found that the state arrested by myricetin corresponds to the hemifusion in proteoliposome fusion. Myricetin blocks C-terminal zippering by binding to the middle region, while allowing SNARE zippering at the N-terminal region. Remarkably, all hemifused vesicles arrested by myricetin were completely converted to full fusion when the myricetin clamp is removed by an enzyme laccase and the fusion reaction was triggered by Ca^2+^. The results imply that the hemifusion is likely to be an on-pathway intermediate in Ca^2+^-triggered exocytosis (Heo et al., 2016). Further, the study raises the strong possibility that the hemifusion is induced by the N-terminal half-zippered SNARE complex.

One might now wonder how hemifusion is possible despite SNAREs being only half-zippered at the N-terminal region. Under these conditions, the SNARE complex would not be forcing apposition of two membranes due to the flexibility at the C-terminal half. Alternatively, the highly basic juxtamembrane regions might play a role here. In fact, electrostatic stitching of two negatively charged membranes by the polybasic sequence has been previously proposed as a potential mechanism for membrane merging (Williams et al., 2009).

Purely energetically speaking, it was recently shown that only one SNARE complex may be sufficient for hemifusion (van den Bogaart et al., 2010; Shi et al., 2012). Partial SNARE zippering generates about 35 *k*_B_*T*, corresponding closely to the energy needed for hemifusion (Li et al., 2007). Half-zippering releases 26 *k*_B_*T* in the presence of membranes and 35 *k*_B_*T* in the presence of the pre-assembled C-terminal domains (Gao et al., 2012; Zorman et al., 2014). Because there are multiple SNARE complexes in the synaptic vesicles (Lang et al., 2001; Takamori et al., 2006), it is possible that hemifusion is efficiently achieved by multiple half-zippered SNARE complexes.

## Hemifusion in the Endocytotic Pathway

**Figure 3:**
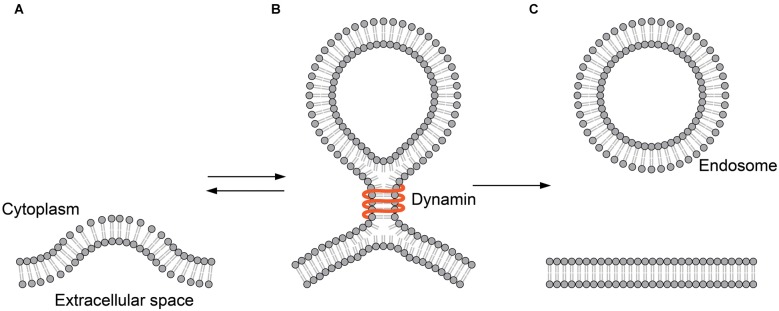
**Hemifusion in endocytosis. (A)** The plasma membrane is invaginated by endocytic machinery such as clathrin. **(B)** Dynamin binds to the neck of the hemifission intermediate formed during endocytosis. **(C)** The membrane sac is detached from the plasma membrane to form an endocytic vesicle.

In the endocytotic pathway, vesicles are created by membrane fission. Membrane fission is a topologically opposite process to membrane fusion. But, this reaction might as well transit through the hemifusion. A GTPase dynamin is the best-studied membrane fission protein. The dynamin protein binds to the neck of the spherical membrane sac bulged from the plasma membrane (Figure 3). The GTP-driven conformational change of dynamin constricts the neck to detach the vesicle from the plasma membrane (Antonny et al., 2016).

In a clever experiment using a tubular membrane capillary, Bashkirov et al. (2008) have shown that dynamin-induced membrane fission is leak-free, representing the fission through hemifusion (Bashkirov et al., 2008). Here, it is clear that dynamins remain on the periphery and are not integral parts of the intermediate. Thus, the role of the protein is clearly defined as the mechanical energy source that drives the remodeling of the membrane.

Recently, hemifusion and hemifission structures were observed in live cells, which provided strong evidence of the hemifusion model against that of a TMD-lined pore. Zhao et al. (2016) observed membrane fusion directly in live chromaffin cells in real time using super-resolution stimulated emission depletion microscopy. They observed a Ω-shaped hemifusion structure in the live cell, adding further evidence that the hemifusion indeed exists along both the fusion and fission pathways.

## Perspectives

Although exocytotic membrane fusion was initially considered to traverse the TMD-lined fusion pore, evidence has accumulated to support an alternative pathway through the lipidic hemifusion and fusion pore. The fusion pathway through the hemifusion appears now to be shared by many membrane fusion systems including viral-cell and intracellular membrane fusion. Not surprisingly, the common hemifusion intermediate is shared by endocytotic membrane fission as well, where no TMD is directly involved in the process. The core feature of the hemifusion is that it is lipidic in nature, although some regulatory participation of TMDs cannot be ruled out. In this model, the proteins may stay at the periphery, corralling lipids at the fusion center to undergo merging. As cryo EM and other imaging methods are making significant strides in improving resolution, we are sure that the mechanistic models described here will be tested in the very near future.

## Author Contributions

D-HK, BK and Y-KS wrote the article. All authors reviewed the manuscript.

## Funding

This work was supported by the National Institutes of Health GM05290 and 5U54GM087519. Korea Healthcare Technology R&D Project, Ministry of Health and Welfare, Republic of Korea (Grant No.: HN14C01010000).

## Conflict of Interest Statement

The authors declare that the research was conducted in the absence of any commercial or financial relationships that could be construed as a potential conflict of interest.
